# *De Novo* Variant in the *RPL27* Gene in a Second Infant with Diamond-Blackfan Anemia

**DOI:** 10.1155/2024/6626388

**Published:** 2024-07-03

**Authors:** Joshua Glass, Julia Weston, Amy Feldman Lewanda, Suvankar Majumdar

**Affiliations:** ^1^ Center for Cancer and Blood Disorders Children's National Hospital, Washington, DC, USA; ^2^ Rare Disease Institute Children's National Hospital, Washington, DC, USA

## Abstract

We describe a 10-month-old female with Diamond-Blackfan anemia (DBA) who presented with macrocytic anemia and reticulocytopenia. Whole exome sequencing revealed a *de novo* intronic variant in *RPL27* (NM_000988.3:c.-2-1G > A p.?) previously reported in one individual with DBA. The existing literature suggests the *RPL27* gene encodes for a ribosomal protein involved in pre-rRNA processing and erythropoiesis. Further research is needed to assess the functional significance of this variant and its implications for genetic testing and therapeutic strategies. This case expands the clinical spectrum of *RPL27*-associated DBA and highlights the importance of reclassifying this gene to likely pathogenic.

## 1. Introduction

Diamond-Blackfan anemia (DBA) is a rare inherited bone marrow failure syndrome that is often characterized by severe macrocytic anemia in the setting of red cell aplasia [1]. The disorder is caused by genetic mutations that are involved in the development and function of the bone marrow. DBA affects approximately seven people per million live births per year [2].

Classical DBA is diagnosed if all four diagnostic criteria are present: onset of anemia at age less than one year, macrocytic anemia with no other significant cytopenias, reticulocytopenia, and a normal marrow cellularity with a paucity of erythroid precursors [2]. Minor criteria include elevated erythrocyte adenosine deaminase (eADA) level activity, congenital anomalies associated with DBA, elevated fetal hemoglobin, and no evidence of another inherited bone marrow failure syndrome. Neutropenia and thrombocytopenia may also be present. Genetic testing can be used to identify mutations in the genes that are known to cause DBA [2]. In about 75% of patients with this diagnosis, there are associated mutations or large deletions in any of 20 ribosomal protein genes [3].

Approximately 60–70% of DBA cases are caused by a pathogenic variant in genes encoding for ribosomal proteins. There are currently 23 different genes known to be associated with DBA-*RPL5*, *RPL11*, *RPS 7*, *RPS 17*, *RPS 24*, *RPS 10*, *RPS 19*, *RPS 26*, *RPL3*, *RPL7*, *RPL14*, *RPL19*, *RPL26*, *RPL36*, *RPL23A*, *RPL35*, *RPS 15*, *RPS 8*, *RPS27A*, *RPL35*, *RPL18*, *GATA1*, and *TSR2* [4, 5]. Two genes, *GATA1* and *TSR2*, follow an X-linked recessive pattern, while the remaining genes follow an autosomal dominant pattern. When a pathogenic mutation is identified, it is inherited from a parent in an autosomal dominant fashion approximately 40–45% of the time. The remaining 55–60% of cases are sporadic and are caused by *de novo* pathogenic variants, as seen in our patient. Up to 30–35% of DBA cases do not have a genetically identifiable cause [5, 6].

In this report, we describe a 10-month-old female with Diamond-Blackfan anemia that initially presented with macrocytic anemia with reticulocytopenia and an elevated erythrocyte adenosine deaminase level. Extended work up revealed a *de novo* variant in the *RPL27* gene (c.-2-1 G> A (p.?)), a candidate gene related to DBA. The variant identified in our patient has only been identified in one other patient, a female diagnosed with DBA diagnosed at birth [7].

## 2. Case Presentation

A 10-month-old female presented to the emergency room after referral from the general pediatrician with concern for poor weight gain and anemia (hemoglobin 3.3 g/dL) found at a well child appointment. This was the patient's first well check since the 4 month appointment, at which time there were no concerns. There was no recent history of fever, cough, congestion, shortness of breath, vomiting, diarrhea, or rash. In addition, there were no bleeding symptoms of bruising, hemoptysis, hematemesis, hematochezia, melena, or any joint pain or swelling, or lymphadenopathy.

The patient is the product of a 41-week gestation delivered via cesarean section due to a failed induction and dropping fetal heart rate. She was brought to the NICU for an aspiration (possible meconium) and stayed about 1 week before being discharged (Table 1). She passed her newborn hearing screen. The pregnancy was planned and naturally conceived. Her mother was on prenatal vitamins, but denied other medication, alcohol, cigarette, or drug use. She denied pregnancy complications such as diabetes, pre-eclampsia, infection, fever, rash, thyroid problems, hypertension, or bleeding. For the entire pregnancy, fetal movements were decreased. She was closely monitored in later pregnancy for this; it was mentioned that the baby seemed to be moving well, but the mother may not have felt it due to larger maternal size. Ultrasound studies were normal. No maternal serum screen, amniocentesis, or chorionic villus sampling was performed.

The patient is the only child born to her parents. The maternal family is from El Salvador, and the paternal family is Black/African-American. Consanguinity was denied. There is no known family history of birth defects, autism, neuromuscular/skeletal issues, recurrent miscarriages, early onset hearing/vision loss, or early onset cancers, anemia, and bleeding or clotting disorders.

The mother's medical history is significant for one early miscarriage in the first trimester; she is otherwise healthy. She has three full sisters; two living sisters are healthy and one sister died shortly after birth for unknown reasons. One sister has a son and daughter who have anemia treated with iron supplementation. The other sister has two healthy children. The mother also has two maternal half-brothers who are healthy and have no children by choice. The patient's maternal grandfather is healthy, and maternal grandmother died before 40 years old from kidney failure, though specifics are unknown.

The father is healthy. He has three brothers and one sister. One of the brothers is an identical twin who has a psychiatric history of bipolar disorder and schizophrenia; he has a son with a seizure disorder. His other siblings are healthy and have healthy children. The paternal grandmother is healthy; there is no information on the paternal grandfather.

The patient was exclusively breastfed, with minimal introduction of solid foods. Aside from a multivitamin, she did not take any other medications. The family reported concern for delayed development milestones, specifically related to gross motor development given the patient's inability to roll over, sit independently, or crawl. She had not been referred for physical therapy. She had previously undergone cardiology evaluation for a murmur, which was determined to be due to an atrial septal defect, and has since had follow-up that demonstrated resolution and normal cardiac anatomy.

Initial evaluation was notable for pallor on exam, but the child was otherwise hemodynamically stable. Subsequent bloodwork revealed a macrocytic anemia with reticulocytopenia (Table 1). Direct antiglobulin testing was negative, and there was low concern for hemolysis, as review of the smear demonstrated large red cells with otherwise normal morphology. A complete metabolic panel, including electrolytes and liver function, was unrevealing. During admission, an abdominal ultrasound was obtained that did neither demonstrate splenomegaly, nor any genitourinary pathology which can be seen with certain hereditary anemias. Nutritional workup revealed a slightly low vitamin B12 level (284 pg/mL (normal range 310–1379 pg/mL)), for which the patient was started on cobalamin supplementation; folate level and iron studies were unremarkable. During the hospitalization, the patient received a total red blood cell transfusion volume of 15 mL/kg with improved hemoglobin levels.

An eADA level prior to transfusion was found to be elevated (1012 mU/g (normal range 400–900 mU/g)). Bone marrow aspiration and biopsy showed normal cellularity (70–80%) with trilineage hematopoiesis with no evidence of hematologic malignancy. A hereditary anemia NGS panel through PerkinElmer Genomics, run through her hematology team, was ultimately nonexplanatory for her clinical diagnosis. This panel encompassed 51 genes that were tested for both sequencing and deletion/duplication variants. These findings included one pathogenic, complex reduced penetrance variant in the *G6PD* gene. This complex variant includes two different gene changes (NM_001042351.1: c.202G > A; 376A > G p.Val68Met; Asn126Asp), with both changes known to be on the same (paternal) chromosome. A homozygous variant in the UGT1A1 gene (c.-55_-54insAT) related to Gilbert syndrome was also identified. Neither of these findings explained her diagnosis.

The patient was seen by genetics at 15 months of age. Due to her unrevealing previous testing, whole exome sequence (WES) trio testing was recommended and ordered through GeneDx Laboratories. WES identified one variant of uncertain significance (VUS) in the *RPL27* gene (NM_000988.3: c.-2-1G > A p.?). As such, a suspected diagnosis of nonclassical DBA was made.

## 3. Discussion

The diagnosis of Diamond-Blackfan anemia has traditionally been made by clinical and laboratory findings. While a diagnosis of DBA typically presents as severe anemia in infancy, the clinical phenotype is remarkably diverse and is characterized by macrocytic anemia, congenital abnormalities, and cancer predisposition. The described patient did not meet the criteria for classical DBA, given the marrow findings with trilineage hematopoiesis. However, a nonclassical diagnosis of DBA could be made in the presence of a DBA-associated genetic variant [2]. This is the likely diagnosis for the patient described in this report, as the mutation found has been previously reported and is currently classified as a candidate related to DBA [7].

The *RPL27* gene is a candidate gene with a potential relationship to the DBA phenotype. This variant was confirmed to be *de novo* in this child (not inherited from either parent). As per the American College of Medical Genetics (ACMG) guidelines from 2015, all variants in candidate genes are to be considered a VUS until additional evidence is reported to support the gene's association with disease [8]. While the existing literature suggests that the *RPL27* gene encodes for ribosomal protein L27, part of the large 60S subunit of the ribosome, the function of *RPL27* gene is not fully understood [4].

The patient identified by Wang et al. presented with cardiac findings, including an atrial septal defect and pulmonary stenosis, and was 2 years old at the time of that report [7]. Our patient was found with an atrial septal defect; given the reported patient's overlap of symptoms and the fact that this variant is confirmed *de novo* in the child, it is likely this gene and variant (NM_000988.3: c.-2-1G > A p.?) are related to this patient's clinical features. Given what is now known when considering the course of these two patients, we propose a mild phenotype associated with this *de novo* mutation.

The variant in the *G6PD* gene was not believed to be related to the diagnosis as it was inherited from her father who has never been symptomatic. While it is true that G6PD deficiency can be seen in heterozygous females, the expression is quite variable. As signs of hemolysis were absent throughout this patient's presentation, we believe that this finding was not contributory to the anemia [9].

For patients older than 12 months that are diagnosed with DBA, glucocorticoids are the first-line treatment option. Given the risk of adverse effects of glucocorticoids in patients younger than 12 months, steroid use is typically deferred in favor of transfusion therapy [2]. As our patient was 10 months old at the time of presentation, she was supported with transfusion therapy. To maintain a recommended hemoglobin level above 8 g/dL, she received additional red cell transfusions at two weeks and then again at six weeks from initial presentation. She has since remained transfusion independent, now more than one year from the initial emergency room visit at the time of this report (Table 1).

If not for genetic testing, it is possible that the diagnosis of DBA would have been missed and the appropriate clinical monitoring and anticipatory guidance could not be provided. Given the increased risk of both hematologic malignancies and solid tumors in patients with DBA, routine follow-up will be important for this patient [10, 11].

## 4. Conclusion

The genetic landscape of DBA remains dynamic. Novel causative genes and mutations continue to emerge, underscoring the complexities inherent to this disorder. Identification of a second patient with a *de novo* variant in *RPL27* suggests that this gene should be considered as pathogenic in future patients undergoing evaluation for this hematologic disorder.

## Figures and Tables

**Table 1:** DBA case report_FINAL61924.

Complete blood count	Value (initial CBC obtained on day of life 1)	Value (at initial presentation)	Value (one year from initial presentation)	Reference ranges
White blood cell count	12.4 K/mcL	12.07 K/mcL	5.73 K/mcL	6.5–13 K/mcL
Hemoglobin	13.1 g/dL	2.8 g/dL	12.4 g/dL	10.2–12.7 g/dL
Platelet count	224 K/mcL	302 K/mcL	287 K/mcL	214–460 K/mcL
Mean corpuscular volume	124.8	111.3 fL	85.1 fL	71.3–82.6 fL
Mean corpuscular hemoglobin concentration	33.8 g/dL	35.4 g/dL	32.3 g/dL	31.9–34.2 g/dL
Red cell distribution width	19.9%	16%	13.5%	12.7–15.1%
Automated reticulocyte %	3.7%	0.7%	0.89%	1.26–2.32%
Absolute reticulocyte count	107.5 K/mcL	5 K/mcL	40.1 K/mcL	57–145.5 K/mcL
